# Validation, visibility, vagueness and variation: A qualitative assessment of existing veterinary guidelines for antimicrobial use in cattle and sheep in the UK

**DOI:** 10.1371/journal.pone.0294733

**Published:** 2023-11-30

**Authors:** Caroline M. Best, Alison M. Bard, Gwen M. Rees, Kristen K. Reyher

**Affiliations:** 1 University of Bristol Veterinary School, Langford, Bristol, United Kingdom; 2 Department of Life Sciences, Aberystwyth University, Aberystwyth, Ceredigion, United Kingdom; Cornell University College of Veterinary Medicine, UNITED STATES

## Abstract

Antimicrobials are essential in veterinary medicine to treat and control bacterial disease in animals. Their prudent use in food-producing animals has been encouraged to reduce the development and spread of antimicrobial resistance. National and international guidelines for responsible antimicrobial use have been developed as tools to guide and rationalise antimicrobial prescribing decisions by veterinarians and usage decisions by farmers. Yet, there is little understanding of whether these existing guidelines are fit for purpose. Accordingly, this study rigorously assessed 128 veterinary guidelines for antimicrobial use in ruminants in the UK, following established qualitative methodologies. Findings revealed four pertinent themes: validation of the veterinarian as the prescriber, visibility of responsible use realities, vagueness in interpretation and variation in directing behaviour. These themes encompassed the roles and responsibilities of the veterinarian and the realities of prescribing scenarios, alongside concerns relating to the specificity within and variation between guidelines. Resultant recommendations to inform and support the future development of guidelines include establishing species-specific and disease-specific guidelines, expanding guidelines to include disease prevention measures, including definitions to resolve vagueness and promoting congruence in interpretation, encouraging the development of practice-level guidelines to endorse collaboration and ownership, and fostering active working between stakeholders to align priorities and messaging.

## Introduction

Antimicrobials play an essential role in veterinary medicine to treat and control bacterial disease in animals [1]. However, antimicrobial use in food-producing animals is under increased scrutiny as any use of antimicrobials can culminate in the development of antimicrobial resistance (AMR) [2]. Whilst the implications of AMR on animal health are significant, the potential for cross-species transmission of resistant bacteria from animals to humans is of grave concern [3]. Accordingly, interventions that restrict antimicrobial use in food-producing animals have received considerable attention in recent years because of growing awareness that reduced antimicrobial use is potentially associated with a reduction in the presence of resistant bacteria in animals [3].

In the UK, veterinary antimicrobials are classed as prescription-only medicines and can only be prescribed by a veterinarian (POM-V) [4]. As gatekeepers to antimicrobial use, veterinarians are increasingly pressured to avoid inappropriate prescribing in order to reduce AMR in bacteria of animal origin. Antimicrobial stewardship initiatives to encourage responsible prescribing are now fairly common in veterinary medicine, with various UK and worldwide organisations producing guidelines for responsible use. Guidelines serve to foster consistent best-practice prescribing by highlighting the overall principles of responsible use and specific recommendations for the selection of antimicrobials in veterinary practice [5]. As a result, guidelines for responsible use are important tools for veterinarians to rationalise antimicrobial use and minimise the development of AMR [1,6–8].

In the UK Government’s five-year national action plan on AMR, the development of guidance for antimicrobial users and prescribers was highlighted to encourage the uptake of recommended best practice and to strengthen stewardship for responsible use [9]. Whilst guidelines can influence prescribing behaviour of veterinarians [10], how guidelines are developed and written influences how often they are used and how they improve clinical practice [11]. Often veterinarians are aware of responsible use guidelines but work independently of them, resulting in variation between individual prescribing practices [12]. This is in contrast to countries like Germany who have compulsory guidelines for prudent use of antimicrobials in animals [6]. Few studies have reviewed existing antimicrobial use guidelines in veterinary medicine; Teale and Moulin [13] demonstrated in a narrative review the variety of guidance provided by different organisations, and Allerton et al. [14] objectively assessed guidelines pertaining to prudent antimicrobial use in dogs and cats in Europe.

Given the number of readily available guidelines, it was considered timely to review existing guidelines in the context of each other and determine whether recommendations can be made for their improvement. To the authors’ knowledge, this is the first study to qualitatively review veterinary guidelines for antimicrobial use pertaining to cattle and sheep in the UK. Using established qualitative methodologies, the aim of this study was to rigorously assess existing responsible use guidelines within the ruminant sector with the intention to construct recommendations to inform and support the future development of guidelines and codes of practice. The focus of exploration was driven by three main research questions: (1) How do guidelines compare in terms of content and quality? (2) How is responsible antimicrobial use conceptualised across guidelines? (3) Where do tensions lie in the practicality and feasibility of guidelines with the aim of directing behaviour?

## Materials and methods

### Study setting

This study was designed to support the development and establishment of a veterinary code of conduct and clinical guidelines for antimicrobial prescribing by the Arwain Veterinary Prescribing Champions network, established within the complex intervention Arwain Vet Cymru (AVC) [15] and continued within the wider Arwain Defnydd Gwrthfioteg Cyfrifol (Arwain DGC) project in Wales. Arwain DGC is an interdisciplinary project built on the pioneering work of AVC seeking to promote the responsible use of antimicrobials in animals. The project was closely aligned with the Welsh Government’s “Five-year Implementation Plan for AMR in Animals and the Environment” launched in 2019, which highlighted the development of robust and consistent guidelines as a key construct of ambitions to optimise the use of antimicrobials in animals [16].

### Reflexivity statement

This study was mainly conducted by the first author (CMB). CMB is a senior research associate within the field of AMR and AMU with a background in veterinary epidemiology (PhD), veterinary pharmacy (MSc) and zoology (BSc). CMB has training and experience in independently conducting qualitative research methodologies, including thematic analysis. CMB has experience in and connections to the cattle and sheep sectors in the UK. All other authors (AMB, GMR and KKR) have experience in qualitative methodologies. GMR and KKR are qualified veterinarians.

### Study design

A systematic method of document analysis was conducted to assess existing guidelines for antimicrobial use, which followed the READ approach of (1) readying materials, (2) extracting data, (3) analysing the data and (4) distilling the findings [17].

#### Document selection

As defined by Dalglish et al. [17], the term ‘document’ was taken to mean “a physical or virtual artefact designed by creators, for users, to function within a particular setting”. In this study, documents were selected based on their provision to inform and advise people on how a specified behaviour or goal relating to antimicrobial use should be achieved (e.g. responsible prescribing of antimicrobials) or what a specified behaviour or goal relating to antimicrobial use should be (e.g. responsible use of antimicrobials). Therefore, the types of documents analysed in this study included a mix of written sources such as guidelines, position statements and policy documents, referred to hereafter as “guidelines”.

Guidelines were identified through internet searches, using the search engine Google without an anonymous virtual private network, to find publicly available online material. Search terms were “antibiotic” or “antimicrobial” and “veterinary”, “guidelines”, “policy” and “position statement”. No additional searches of non-electronic guidelines were conducted. All guidelines published since January 2013 were eligible, given that guidelines have evolved significantly over time as a result of the shifting political and scientific landscape. However, not all guidelines were dated and these were included at the researchers’ discretion. If more than one version of any guideline was available, only the latest update (if known) was included. Searches were limited to stakeholders based in the UK, however some international guidelines were also included because of their potential influence on UK prescribing practice. Only guidelines published in English were included. Guidelines were displayed as either Portable Document Formats (PDFs) or webpages. Guidelines were purposively selected for their relevance to antimicrobial use and prescribing in food-producing animals, in particular cattle (dairy and beef) and sheep, given these foci were central within the Arwain DGC project. Thus, guidelines specifically pertaining to other species, such as poultry and pigs, were excluded from selection. Both veterinarian- and farmer-directed material were included due to the consensus that guidelines aimed at farmers would impact antimicrobial prescribing by the veterinarian.

#### Data extraction

Content analysis was employed as the process of organising information into categories related to the central research questions [18], before taking an overall thematic exploration to develop themes across the data set. First, guidelines were read and examined by the first author (CMB) to identify meaningful and relevant sections of text. This text was extracted and collated in Microsoft Excel, with rows representing the stakeholders which published the guideline and columns with umbrella categories of data content. Data extraction and collation were performed iteratively alongside data collection, meaning that guidelines were revisited in response to developing and expanding umbrella categories. Searches for documents ceased at the point of saturation, when additional searches identified no new material. Importantly, this study did not seek to analyse a definitive set of all available guidelines but instead aimed to capture a highly varied dataset of material.

#### Data analysis

Reflexive thematic analysis [19] was conducted in full by the first author (CMB) to qualitatively analyse and identify the themes across extracted data. Data were read and re-read before performing manual line-by-line coding in Microsoft Word. Coding identifies and provides a succinct label for a feature of the data that is relevant to the research questions [20] and is an “inherently subjective process, one that requires a reflexive researcher–who strives to reflect on their assumptions and how these might shape and delimit their coding” [21]. In line with reflexive thematic analysis [19], coding was performed by a single coder (CMB). Coding was inductive (data-driven) and included both descriptive (semantic) and interpretative (latent) codes. Codes were then categorised and developed into themes. Themes are “creative and interpretive stories about the data, produced at the intersection of the researcher’s theoretical assumptions, their analytic resources and skill, and the data themselves” [22]. Theme development was an organic and active process, which was closely scrutinised to ensure that themes conveyed the richness and complexity of data, whilst remaining representative of the entire data set.

Conceptualised themes were shared and critically discussed with the other authors as part of the analytic process to enhance reflexivity and interpretive depth, rather than seeking consensus between analysts [19]. Reflexive thematic analysis fully embraces the subjective skills of the researcher, where “a research team is not required or even desirable for quality” [19]. Therefore, the other authors provided input through informal discussions of the themes as they unfolded, given their experience and familiarity of the topic area, but did not override the construction of themes. Quantitative measures of coding accuracy (e.g. intercoder reliability) were not considered aligned to the practice of reflexive thematic analysis, as here: “researcher subjectivity is conceptualised as a resource for knowledge production, which inevitably sculpts the knowledge produced, rather than a must-be-contained threat to credibility” [19].

## Results and discussion

Antimicrobial use guidelines were produced by a diverse range of stakeholders. This reflects awareness that antimicrobial stewardship is a shared duty across a normative chain of responsibility from ‘farm to fork’, but also a result of the intensified interest with respect to food security and governance of antimicrobial use in UK food-producing animals. In total, 128 unduplicated veterinary guidelines on antimicrobial use produced by 10 stakeholder groups were reviewed in this study (Table 1). All guidelines are listed in S1 Table. The date range of dated guidelines spanned between 2012 and 2023, with the majority produced between 2017 and 2023.

**Table 1:** The number of guidelines for antimicrobial use reviewed from eFach stakeholder group.

Stakeholder group	Number of guidelines reviewed
Veterinary, animal health and medicine organisations	41
Milk and meat processors	20
Government organisations and governing bodies	27
Retailers and restaurants	18
Food standards organisations and collaborations	4
Cross-sector working groups and partnerships	2
Levy boards, cooperatives and associations	8
Farm certification bodies	6
Farming unions	1
Farm and business support	1
**Total guidelines reviewed**	**128**

### Content analysis

There was considerable variation in the quality, scope, direction and comprehensiveness of guidelines produced by stakeholders. However, despite the large number of voices involved with potentially different agendas at play, guidelines shared a common narrative and goal of encouraging responsible antimicrobial use practices to reduce pressure on the development of AMR.

**Fig 1:**
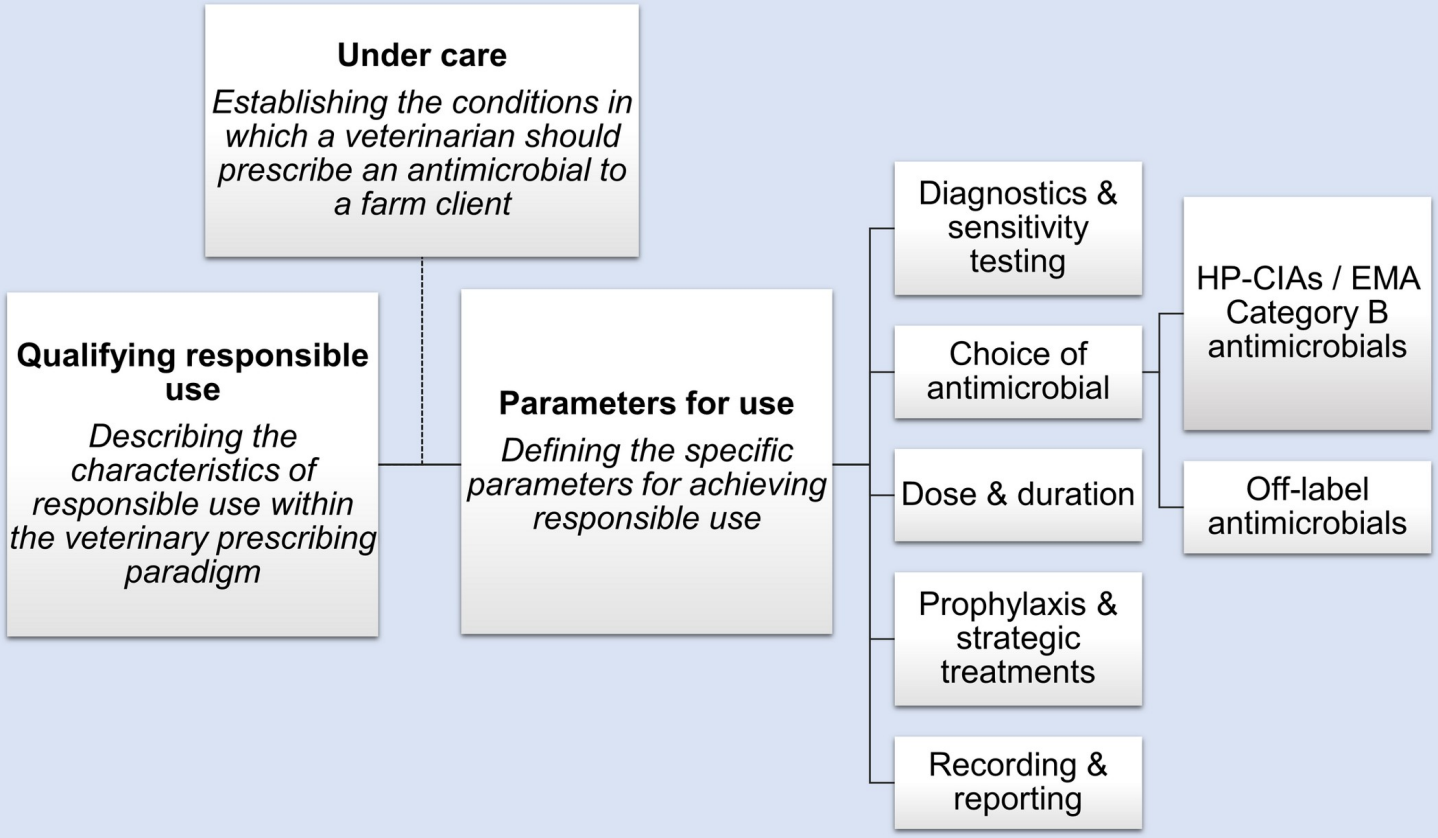
Core components of antimicrobial use guidelines identified by content analysis. HP-CIAs: Highest Priority Critically Important Antimicrobials as defined by the World Health Organization (WHO); EMA: European Medicines Agency (EMA).

Guidelines were shaped by a number of common principles which attempted to outline and describe the conditions for responsible antimicrobial use. Firstly, guidelines set out their intention by framing their defining characteristics of responsible use within the context of minimising AMR, preserving the efficacy of existing antimicrobials, and safeguarding animal and human health. Most guidance then explored the technical parameters of responsible use within defined areas of diagnostic practice and treatment decision making. Some guidance also established the conditions in which a veterinarian should prescribe an antimicrobial to a farm client, referring to the principles of “under care”. A summary of the overarching guideline content is outlined (Fig 1).

### Thematic analysis

**Fig 2:**
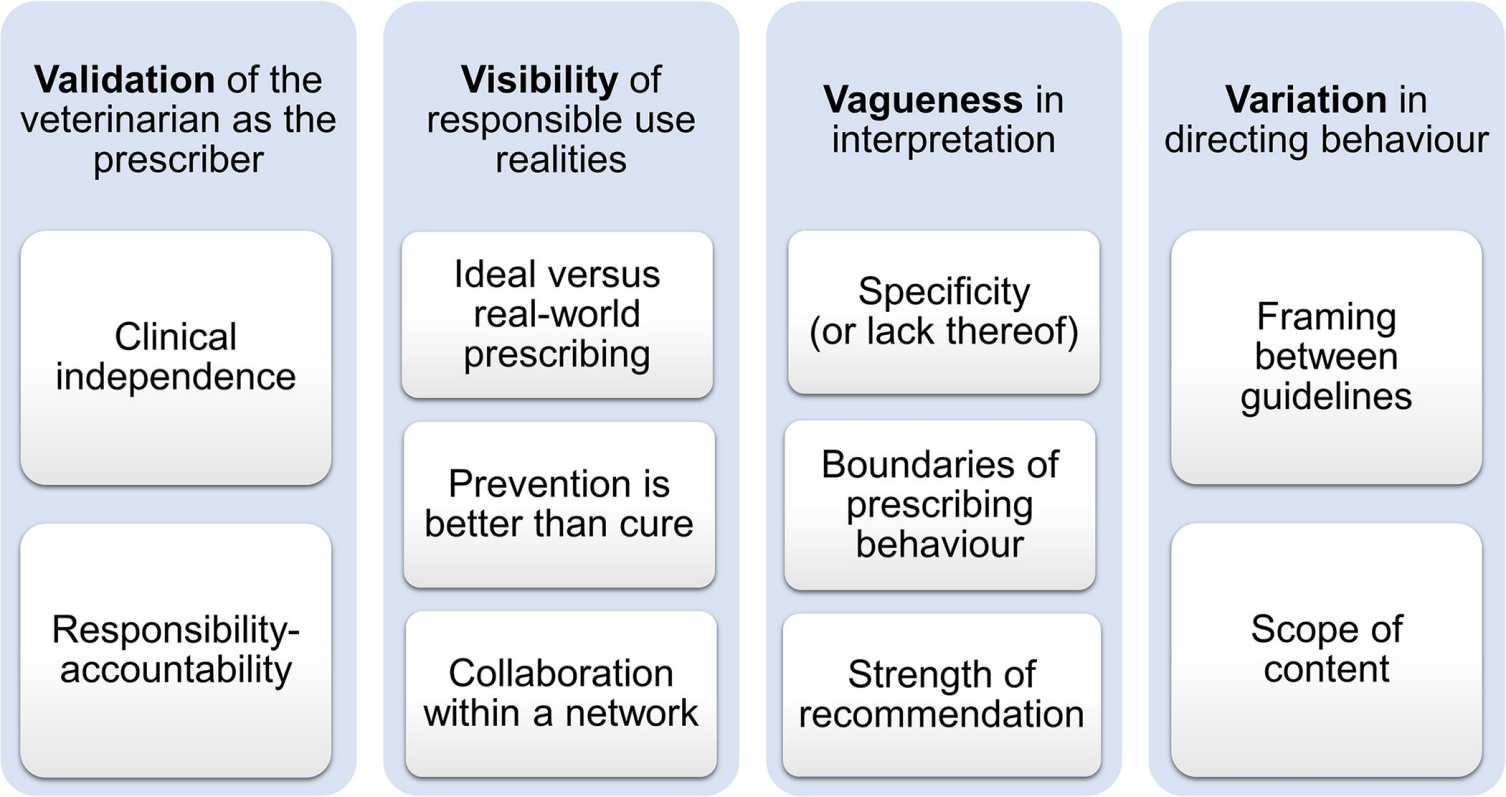
Themes and subthemes of antimicrobial use guidelines identified by thematic analysis.

Based on the information presented in the various antimicrobial use guidelines, four broad themes relating to the research questions were constructed: (1) Validation of the veterinarian as the prescriber, (2) Visibility of responsible use realities, (3) Vagueness in interpretation, and (4) Variation in directing behaviour. Each theme was underpinned by subthemes which captured an important facet of the overarching concept. The themes and subthemes are summarised (Fig 2).

#### Theme 1: Validation of the veterinarian as the prescriber

This first theme encompasses the way in which guidelines demonstrate, support and affirm the roles and responsibilities of veterinarians as key actors within the responsible use of antimicrobials.

#### Clinical independence

Guidelines validated and emphasised the identity of veterinarians as professional guardians of antimicrobials. This narrative highlighted the professional autonomy of the veterinarian, emphasising that decisions to prescribe antimicrobials ultimately rested with the prescribing veterinarian. Here, veterinarians were encouraged to select the most suitable antimicrobial using their professional judgement:

“*Professional judgement is used to decide if an antibiotic is required and if so*, *which antibiotic should be given*.” [23]

Many guidelines referred to the importance of veterinarians’ professional judgement in independent decisions to prescribe antimicrobials. For most guidelines, professional judgement was the conclusion at which a veterinarian arrives following a synthesis of available information from an accurate diagnosis, diagnostic testing results and their own clinical experience. Acquiring knowledge from antimicrobial sensitivity testing (AST) was encouraged in some guidelines as a further tool to inform and support decision making as part of a risk-based assessment. AST plays an important role in the decision-making process when prescribing antimicrobials [24]. However, the time lag between sampling and obtaining the result is often a barrier to its use in practice [25] in addition to sampling difficulties experienced on farm. Therefore, some guidelines implied that decisions to conduct AST were themselves based on professional judgement:

“*The clinical judgement of the veterinarian regarding the need for* [sensitivity] *testing is of paramount importance*.” [26]

Some guidelines promoted veterinarians’ knowledge of the farm and the individual clinical case as important factors informing decision making. Accordingly, guidelines emphasised the importance of veterinarians having knowledge of the specific disease context on farm, taking into consideration disease epidemiology:

*“The prescribing veterinarian should have a good knowledge of the epidemiology of disease on the farm (e*.*g*. *virulence of organisms) and the risk factors for infection associated with the group*, *e*.*g*. *the immune status*, *management factors*.*”* [27]

Knowledge of the farm is an underpinning component of clinical judgement and autonomous decisions to prescribe antimicrobials, reflected in the term “under care” defined within the Royal College of Veterinary Surgeons (RCVS) Code of Professional Conduct [28]. Ensuring comprehensive knowledge of the farm is an important outcome of the one-to-one farmer-veterinarian relationship found in The Netherlands [29] and this on-farm knowledge is considered a precious resource [25]. Farmers also consider their farms to be geographically, physically and biologically unique, and express a need for treatment decisions to be tailored with the uniqueness of their farm [30]. Encouraging veterinarians to use their knowledge and clinical judgement is likely to fulfil farmers’ expectations of veterinarian decision making. Clinical experience was also an underlying construct of independent prescribing decisions. Guidelines highlighted clinical experience to complement decisions to use antimicrobials:

*“The decision to use antimicrobial agents should be based on sound clinical judgement*, *experience*, *and treatment efficacy*.*”* [31]

Whilst clinical experience is an important factor influencing veterinarians’ choice of antimicrobial [32], over-reliance on clinical experience may negatively impact prescribing behaviour by presenting a barrier to the change of habitual practices [33], and uptake of responsible use recommendations [32]. Here, veterinarians can value their autonomy and knowledge beyond that of prescribing protocols, ultimately disregarding them [34]. Similarly, veterinarians prefer to rely on their own observational and clinical skills and experience, rather than using diagnostic tests [35]. As such, fear of losing clinical autonomy could act as a barrier for the implementation of responsible use protocols and recommendations within practice [34]. In order to foster synchronous prescribing decisions, guidelines need to respect and maintain individual autonomy by giving veterinarians freedom of choice, although this will require guidelines to strike the balance between encouraging clinical freedom and becoming too vague. Prioritising freedom of choice in guidelines could act as a license for veterinarians to disregard guideline recommendations inappropriately.

Some guidelines made attempts to support independent decision making by directing veterinarians to consult guidelines from other organisations, including the European Medicine Agency (EMA) Categorisation of Antimicrobials, the World Organisation for Animal Health (WOAH) list of antimicrobial agents of veterinary importance, and guidance from the Responsible Use of Medicines in Agriculture (RUMA). This may be the result of stakeholders attempting to future-proof guidelines in the ever-changing landscape of antimicrobial policy, although in doing so, may date the recommendations further. Additionally, some guidelines encouraged veterinarians to administer antimicrobials in accordance with the Summary of Product Characteristics (SPC). Whilst the SPC is an important source of information to help veterinarians to prescribe veterinary medicines, a study from 2013 reported veterinarians to generally refer to product labels or client information leaflets rather than SPCs [32]. Guidelines should direct veterinarians to the consolidated list of SPCs provided in the Veterinary Medicines Directorate (VMD) Product Information Database [36].

Participation in continuing professional development (CPD) was also encouraged to bolster veterinarians’ clinical understanding. Provision of CPD with a focus on antimicrobials is a likely way to influence changes in prescribing behaviours [32]. However, time pressures in practice and lack of access to CPD may mean that opportunities to develop knowledge and change prescribing practices are limited in reality [37].

#### Responsibility-accountability

Guidelines reaffirmed the responsibility of veterinarians as antimicrobial stewards. Veterinarians are uniquely positioned as guarantors of the responsible use of antimicrobials in livestock farming [38]. Guidelines placed strong emphasis on the accountability of veterinarians to define the conditions of responsible use. This included the choice of antimicrobial, dosage regimen and route of administration. Both veterinarians and farmers alike place the burden of responsibility for the prudent use of antimicrobials on the veterinarian [39]. Furthermore, veterinarians were considered responsible for precisely defining the exceptional circumstances at which strategic treatments, including prophylaxis, were warranted:

*“Preventive treatment with antibiotics in animals should only be applied to animals diagnosed at high risk of bacterial disease on the basis of epidemiological and clinical knowledge from the prescribing vet*.*”* [40]

Responsibility to justify treatment decisions was also considered a key component of professional practice. Guidance emphasised that veterinarians were required to take ownership and responsibility for their own prescribing decisions:

“*Veterinary surgeons must be seen to ensure that when using antimicrobials*, *they do so responsibly*, *and be accountable for the choices made in such use*.” [41]

Emphasis was placed on the obligation of veterinarians to explain and justify decisions in order to uphold transparency, integrity and accountability, especially when deviating from protocols. As such, some guidelines emphasised that veterinarians’ knowledge was an important element in their ability to justify optimal treatment decisions:

*“Veterinarians must be knowledgeable about antimicrobial availability*, *AMR*, *alternatives to antimicrobials*, *and susceptibility testing*, *because they are accountable for the safe and effective use of these medicines*.*”* [26]

Some guidelines encouraged veterinarians to record written justifications as a means to actively audit the decision-making process. Results of diagnostic and sensitivity testing were frequently recommended as evidence to defend decisions made by veterinarians, particularly for antimicrobials most relevant to human health:

*“Appropriate use of microbiological testing is the use of results from culture and sensitivity test results*, *and other appropriate sensitivity testing methods to justify the use of a medically important antimicrobial*.*”* [42]

Such confirmation of diagnosis can be useful to veterinarians in justifying the choice of antimicrobial–particularly critically important antimicrobials–and associated costs of treatment to the farmer [35]. Veterinarians feel a strong burden of responsibility to ensure that the correct antimicrobial is prescribed [39]. Results from AST can also support veterinarian decisions, reducing disputes with the animal owner [24].

Responsibility of veterinarians also extended to the review of prescribing decisions, as a mechanism to validate or refine choices made. Guidelines highlighted that prescribing decisions are not set in stone and should be reviewed on the basis of clinical outcomes or microbiological tests if antimicrobials are prescribed in the interim between obtaining results.

#### Theme 2: Visibility of responsible use realities

This theme illustrated the awareness and active–although not highly nuanced–recognition of the opportunities and practical realities surrounding the promotion of responsible antimicrobial use in guidelines.

#### Ideal versus real-world prescribing

Whilst guidelines emphasised the broader aims of responsible antimicrobial use–to reduce antimicrobial use in order to minimise the development of AMR and maximise treatment efficacy–guidelines also highlighted the important role that antimicrobials play in safeguarding animal health and welfare:

*“Responsible use means allowing veterinarians to exercise their professional judgement to decide the most appropriate treatment–including antibiotics*. *Without antibiotics*, *disease will spread and animals will suffer*.” [43]

Guidelines reflected that antimicrobial stewardship is not a homogeneous intervention and instead acknowledged the complexity of prescribing decisions present in real-world clinical scenarios. Veterinarians’ prescribing decisions are influenced by a range of clinical and non-clinical factors, including clinical uncertainty, underlying herd/flock health, infrastructure quality, financial pressures, variability in farmers’ personalities and the need to manage relationships with clients and colleagues [44]. As such, recommendations were sufficiently flexible and dynamic to allow veterinarians to use their clinical independence and autonomy, and were framed around the concept of balancing the benefits of treatment with the risk of AMR development:

*“Responsible use of antimicrobials by veterinarians can play an important role in protecting animal and human health*, *and minimise environmental impacts*.*”* [26]

Recommendations within both national and international guidelines were often accompanied by the adverb “*ideally*”, emphasising that guidance reflected stewardship ideals or gold-standard practices which were not always appropriate or feasible in all clinical scenarios. Deviations from responsible use ideals were also qualified by phrases such as “*wherever possible*” and “*whenever possible*”, further highlighting the voluntary nature of recommendations and attempts to accommodate clinical reality:

“*Whenever possible*, *microbiologic diagnosis*, *including culture and antibacterial sensitivity testing*, *should be used to make treatment decisions … Antibacterial susceptibility should ideally be ascertained before therapy is started*.” [26]

This level of flexibility supports views that guidelines should never be a substitute for clinical discretion and judgement and should not dictate standards of practice [45]. Previous research has found veterinarians to place greater importance on their clinical judgement than on guidelines when selecting antimicrobials to prescribe [46]. Clinical judgement is required to adjust and adapt prescribing behaviours to suit individual clinical cases.

#### Prevention is better than cure

Guidelines highlighted that antimicrobials were not an alternative to poor animal husbandry or management, and should not be considered a replacement of best practice on farm:

*“Antibiotics must not be used to compensate for poor hygiene or inadequate husbandry conditions or where improvements in animal husbandry could reduce the need for antibiotic treatment*.*”* [47]

Here, the importance of disease control and prevention as long-term tools to reduce or replace antimicrobial use on farm was emphasised. Certainly infection control and disease prevention play an important role in antimicrobial stewardship [48,49]. Disease prevention measures considered in guidance included good management, nutrition (including prebiotics and probiotics), colostrum, vaccination, biosecurity and farm hygiene:

“*Replace the use of antimicrobial agents in animal agriculture where possible*, *with sustainable solutions to prevent diseases such as vaccination and improved husbandry practices*, *to protect animal health and welfare*.” [50]

In particular, disease prevention and management strategies were highlighted as important tools to reduce the need for prophylactic treatments. Although prophylaxis is now being phased out, in some older guidelines, prophylactic antimicrobial treatment was considered an interim measure whilst alternative prevention strategies were implemented:

*“Prophylactic use of antibiotics is to be avoided wherever possible without compromising animal welfare*. *Where it is used*, *it should be regarded as an interim measure whilst alternative management and vaccination strategies are implemented*.*”* [51]

The importance of disease prevention within health plans was also emphasised in guidelines, playing an essential role in antimicrobial stewardship:

*“Prevent disease by drawing up a herd health plan with your vet which includes routine preventative treatments (e*.*g*. *routine foot care*, *mastitis*, *vaccination and worming)*.*”* [52]

Whilst guidelines outlined suggested strategies for disease prevention, recommendations were not behaviourally specific nor did they provide sufficient detail for measures to be practically implemented. For example, whilst vaccination can lead to significant reductions in the use of antimicrobials [53], explicit detail on available vaccination strategies was rarely included in guidelines. This could be explained by the absence of species-specific clinical guidelines and the ever-changing livestock vaccination landscape. In order for future disease-specific guidelines to be stand-alone support tools, therefore, greater attention must be paid to the role of preventive medicine in responsible antimicrobial use.

#### Collaboration within a network

Whilst the responsibility to prescribe antimicrobials fell to the individual veterinarian, some guidelines highlighted the reality of prescribing within a network of responsibility. Developing written practice guidelines was encouraged to ensure unity in decision making across veterinarians within practice, especially in order to:

“*Promote awareness of AMR more especially through Veterinary Statutory Bodies and Veterinary Education Establishments to encourage a professional culture that supports the responsible and ethical use of antimicrobial products in animals*.” [54]

Within such a network, collaboration between veterinarians was considered important and could be accomplished by, for instance, creating practice-based protocols covering common infections and the circumstances at which strategic treatments (such as metaphylaxis) are considered appropriate. Existing guidelines, particularly international guidelines, appeared typically as general recommendations, presenting an opportunity for veterinarians to come together to agree and develop more comprehensive and relevant working guidelines for practice which are based around the principles of responsible use [55,56]. This includes the incorporation of regional and local trends in disease and AMR. Collaboration was also extended by one stakeholder to highlight the need to agree on a definition of “*under care*” by veterinarians within practices.

“*Agree interpretation of “under direct clinical care” among vets in practice*.” [57]

Guidelines also called for veterinarians and farmers to work together towards the shared goal of responsible antimicrobial use. Accordingly, veterinarian-directed guidance framed the importance of the veterinarian working with the farmer to reduce antimicrobial use in order to meet industry targets:

*“Veterinary surgeons should familiarise themselves with the targets for their sector and work with producers to achieve these targets*.*”* [58]

Many farmers and veterinarians are open to collaboration to achieve prudent antimicrobial use, creating a sense of shared responsibility in reducing antimicrobial use [59]. Some guidelines emphasised that veterinarians were also responsible for promoting the ethos of responsible use to farmers, by providing clear labels and sound technical advice, particularly on withdrawal periods. This was in part due to some stakeholders seeing farmers as an extension of the veterinarian, and recognised that ultimately the farmer makes the final decision as to whether administration instructions are adhered to or not:

“*The use of therapeutic antimicrobials must be under the direction of veterinary surgeons*. *Farmers*, *however*, *have a very important role to play in ensuring that the directions of the veterinary surgeon are properly carried out*.” [58]

Interestingly, some UK-based farmer-directed guidelines highlighted that farmers’ roles also included the responsibility for assessing clinical presentations and delivering the most appropriate antimicrobial treatment, in accordance with veterinarian guidance (e.g. health plans):

“*Antibiotic therapy is only used if there is evidence that shows the need for the treatment—either a vet diagnosis is in place or vet protocols are followed that lead to a diagnosis*.” [60]

“*We expect our farmers to select a* [antimicrobial] *treatment appropriate for the animal and do this in a timely manner in accordance with veterinary guidance*.*”* [61]

One way of facilitating disease control on farms is through health planning [62]. Guidelines highlighted that health planning was essential for antimicrobial stewardship, but required collaboration with the veterinarian and farmer working together to review and achieve responsible use of antimicrobials.

#### Theme 3: Vagueness in interpretation

This theme describes the lack of preciseness in how guidelines communicated recommendations, in terms of the detail provided and the strength of obligation. The authors acknowledge that this is an inevitable consequence of the lack of species-specific clinical disease guidelines relating to common diseases of cattle and sheep. Nonetheless, it is important to highlight these weaknesses to inform their future development.

#### Specificity (or lack thereof) within guidance

Lack of specificity was an underlying weakness of the majority of guidelines reviewed. Responsible use was discussed broadly around the ethos of:

“*As little as possible*, *but as much as necessary*.” [63]

Although some variations of this existed, including:

“*As little as possible*, *but as often as necessary*.” [64]

*“As little as possible*, *but as much as needed*.” [65]

Whilst these phrases succinctly qualify the principles of responsible use, they lack specificity for definitive interpretation in relation to any particular level of antimicrobial use, thus leaving the judgement and decision-making to the individual veterinarian. Some stakeholders built their guidance around the 3Rs framework, originally developed as guiding principles for use of animals in research, which provide greater detail of the key concepts of responsible use:

*“REDUCE the annual usage of antimicrobial agents in animal agriculture*, *per unit of livestock produced (mg/PCU)*, *whilst preserving animal health and welfare… REPLACE the use of antimicrobial agents in animal agriculture where possible*, *with sustainable solutions to prevent diseases such as vaccination and improved husbandry practices*, *to protect animal health and welfare… REFINE the use of antimicrobial agents in animal agriculture*, *by ensuring the responsible and informed selection and administration of products to animals that have a clinical indication for treatment*.*”* [50]

In some cases, stakeholders replaced “*refine*” with “*rethink*” to encourage the reflection of existing prescribing practices and consideration of alternative opportunities such as disease prevention or infrastructure solutions. Nonetheless, ensuring the responsible and informed selection of antimicrobials was an essential component of *refining* use of antimicrobials, as framed by the 3Rs concept in some guidelines, with emphasis on the veterinarian to:

*“Choose the right drug for the right bug*.” [58]

Despite this, there was a lack of actionable recommendations and specific detail, limiting the capacity of guidelines to inform veterinarians’ decision making. These blanket recommendations seldom recommended 1^st^, 2^nd^ or 3^rd^ choice antimicrobials. Listing of 1^st^, 2^nd^ and 3^rd^ choice antimicrobials for common diseases is considered effective in reducing and refining antimicrobial use by veterinarians [66]. Lack of clear guidelines for treating conditions is a barrier to appropriate antimicrobial prescribing practice by veterinarians [67]. When guidelines are considered practical, veterinarians are more likely to prescribe 1^st^ line active substances, compared to when considering guidelines to be impractical [68]. Furthermore, implementation of disease-specific antimicrobial use guidelines can result in more appropriate prescribing of 1^st^ choice antimicrobial treatments [69] and reduced use of highest priority critically important antimicrobials (HP-CIAs; now classified by the EMA as Category B “Restrict”) [70].

Understandably, national guidelines for responsible use of antimicrobials are unable to be sufficiently specific to be applied to all clinical scenarios, but have scope to be much more specific than international guidelines. As such, the deliberate vagueness observed in current guidelines was a product of the multiple clinical conditions and species in which the guidelines must target (and resultant lack of consensus on choice of antimicrobial), in addition to changing licensing, fluctuating stock availability and efforts to honour clinical freedom. Variability among clinical cases means that guidelines must be considered to form the basis of decision making, rather than standards of care that must be followed in all circumstances [56].

Specific dosage and treatment regimens were also lacking in guidelines, despite recommendations highlighting the importance of prescribing the correct dosage regimen. Here, very few guidelines offered any specific dosage (e.g. mg/kg) necessary to ensure the therapeutic efficacy of the antimicrobial agent, nor an average or expected duration of treatment and inter-dosing intervals. However, inclusion of specific dosages is likely to out-date guidelines faster; instead, guidelines should encourage veterinarians to consult the SPC. As such, some guidelines just alluded to the prescribing of antimicrobials over a preferably short time frame, in line with responsible use principles:

*“Therapeutic antimicrobials should be used for as long as needed but for the shortest duration necessary*, *and at the appropriate dosage*.” [26]

Formularies outlining the most appropriate antimicrobials for use in veterinary care in companion animals have been developed [71], but, at the time of writing, none are available for farmed livestock in the UK, despite medicine use in farmed livestock being governed under much stricter control. Whilst the development of a formulary would be valuable for farm veterinarians, the practicalities of its development make it too difficult to implement currently.

Since the purpose of guidelines is to guide behaviour, failing to offer explicit recommendations provides little benefit to veterinarians who are uncertain how to proceed when faced with a clinical scenario. Therefore, guideline developers should be mindful of how recommendations are given, ensuring that, if possible, they are sufficiently specific to provide the greatest clarity of behavioural instructions possible [72]. Recommendations with a specific goal in human health, such as, “Walk 1 mile during lunch three times this week”, have been shown to lead to higher performance compared to those with a general goal, such as, “Exercise more often” [73]. However, the more detailed and specific the recommendations, the longer the guideline is likely to be. Guidelines that are too long may result in key recommendations being unintentionally disregarded [74].

#### Boundaries of prescribing behaviour

There was also a lack of clarity regarding the confines of responsible prescribing, leaving some definitions open to broad interpretation. For example, some stakeholders appeared to distinguish between “*routine prophylaxis*” and “*prophylaxis*”, without providing any definition to differentiate the two practices. Similarly, the conditions for which prophylaxis or metaphylaxis were considered “*appropriate*” or “*inappropriate*” were rarely accompanied by a practical definition. Stakeholders should provide greater clarity in how these practices are defined. The existing lack of specificity in responsible use guidelines likely results in ambiguity and inconsistent interpretation by readers [75], which may hamper applicability and implementation [76].

Lack of specificity was also seen in definitions of the phrase “under care”. Most veterinarian-targeted guidance made reference to prescribing antimicrobials in accordance with the definition of “under care” provided in the RCVS Code of Professional Conduct [28]. Guidance to veterinary professionals on “under care” from the RCVS has recently been revised following public consultation in response to concerns of the robustness of guidance and resultant safeguarding of animal health and welfare [77]. Farm veterinarians across Wales who did not consider the current definition of “under care” fit for purpose have also raised concerns about it leading to an unsustainable situation where farmers are sourcing medicines from multiple different veterinary practices [78]. This introduces complications into calculating antimicrobial use on farms as well as hindering antimicrobial stewardship efforts if clear communication between veterinarians is not in place.

In contrast, some guidelines instructed the prescribing of antimicrobials only to animals “*under veterinary supervision*”, “*under their direct care*”, “*under their direct clinical care*” or “*under veterinary care within a valid veterinarian-client-patient relationship*”; some suggested prescribing only in instances where the veterinarian had “*visited the premises in which the animal is kept sufficiently often and recently enough*”. No guidelines extended beyond this to define “*recently enough*” and “*often enough*”, nor provided specific examples of scenarios which were not considered to be aligned with “under care”. Given that previous definitions of “under care” provided by the RCVS have been perceived as problematic by the industry [79], it is no surprise that stakeholders have been unable to include appropriate definitions to support these key prescribing conditions. Providing real-life examples of prescribing challenges and scenarios can be an effective tool to influence prescribing behaviours [32].

#### Strength of recommendations

There was a lack of certainty and distinctness in the characterisation of recommendation strength and subsequent level of obligation in the guidelines reviewed. A range of modal verbs (“*must*”, “*should*”, “*may*”) were used as deontic terms to express three discrete levels of obligation. “Must” is imperatival and conveys the highest level of obligation with the least amount of variability [80]. Accordingly, “must” or “must not” were often limited to situations where there was a clear instruction to direct mandatory behaviour, such as with regards to the use HP-CIAs:

“*The use of Highest Priority Critically Important antibiotics must be a last resort and their use must be under the direction of a vet*, *backed up by sensitivity or diagnostic testing*.” [81]

“Should” was the most widely used modal verb within the guidelines. It acted as an intermediate level of obligation, portraying a normative recommendation that was desirable but not mandatory, and facilitated flexibility in prescribing behaviours:

“*Ideally*, *preventive administration of antimicrobials should not be used*, *and should certainly not be used routinely*.*”* [82]

The use of “should” in recommendations is likely to result in greater levels of variation in the interpretation of perceived obligation. The RCVS has claimed that replacing “should” with “must” in their Code of Professional Conduct was the key change in language necessary to achieve clearer direction of obligations [83]. However, “should” is considered more appropriate in scenarios where the veterinarian is required to weigh duties to arrive at a considered moral judgement or clinical decision [83]. Therefore, providing flexibility via normative language may prevent unnecessary dilemmas when clinical scenarios are not straightforward and is an appropriate choice to convey an intermediate level of obligation [80]. Antimicrobial prescribing, after all, is considered variable, shifting and adaptable to a given context, regardless of guidelines [84], particularly given guideline adherence is not legally binding.

“May” was also employed in guidelines, despite it conveying the lowest levels of obligation [80]. This may have implications on the uptake of recommendations, such as using antimicrobial sensitivity trends to guide prescribing decisions:

“*Antimicrobial sensitivity trends should be monitored over time and such monitoring may be used to guide clinical judgement on antimicrobial usage*.” [58]

Stronger recommendations for the use of antimicrobial sensitivity testing in guidelines has been shown to increase its uptake in practice [24], however weak recommendations could be interpreted by veterinarians as being the result of poor supportive evidence or ambivalence, which might be perceived as uncertainty regarding the correct recommendation [85]. Furthermore, weak recommendations require greater investment in interpretation and resultant decision making, compared to strong recommendations [85]. On the other hand, veterinarians value their autonomy to prescribe antimicrobials [34] and strong recommendations within guidelines could result in resistance to implementing guidance.

Other constructions of deontic language included phrases such as “*we recommend…*” and “*we suggest…*”. These modified phrases in the passive voice appeared frequently across the guidelines studied and emphasised the descriptive rather than prescriptive nature of guidelines. Indeed many veterinarians consider guidelines to be primarily descriptive due to their generic nature [68,86]. Paying greater attention to the language used within guidelines is essential to strengthen the connection between wording and their intended level of obligation and resultant compliance [87].

#### Theme 4: Variation in directing behaviour

This final theme encapsulates the marked variation and malalignment observed in how guidelines direct responsible use behaviour–despite sharing a common ethos–and the scope of guideline content provided. Guidelines mostly covered broadly similar topics, although there was considerable variation in the quality, scope and breadth of recommendations, suggesting that the perspectives and priorities of stakeholders were not always aligned.

#### Framing between guidelines

Differences in the focus of responsible use guidelines were evidenced between guidelines. Some guidelines centred responsible use recommendations around the absolute or overall reduction in the amount of antimicrobials used. Here, some referred to the 4Rs framework, identifying the requirement to “*record*” use of antimicrobials in order to monitor usage within the supply chain. In comparison, others characterised responsible use as the need to reduce the “*unwarranted use of antimicrobials*”.

Specific recommendations also varied between stakeholders, with some retailer groups having more stringent guidance, such as only permitting use of HP-CIAs (or EMA Category B antimicrobials) as a last resort and prohibiting prophylactic treatments. Lack of consensus in how responsible use is framed across guidelines could reduce the strength of individual recommendations made, resulting in professional ambivalence towards guidelines. However, despite the distinctions in recommendations pertaining to responsible use, there was congruence across guidelines of the underpinning argument of responsible use: to safeguard the future use of antimicrobials in the interests of maximising animal and human health.

Marked nuances in the language of responsible use were also apparent. Guidelines employed a variety of adjectives—often used interchangeably and eliciting different meanings—to describe the intended use of antimicrobials. These included “*prudent*”, “*limited*”, “*judicious*”, “*ethical*”, “*considered*”, “*rational*”, “*targeted*”, “*appropriate*” and “*cautious*”. The breadth of descriptions of antimicrobial stewardship has expanded over time, with movement away from technical descriptions of drugs and dosages to concepts of responsibility [88]. Terms such as “appropriate” or “rational” do not explicitly consider the need to balance individual and societal needs, playing down the inherent value judgement implied by “responsible” [88]. Variation in terminology may result in differences in interpretation, with prescribing practices becoming aligned with veterinarians’ individual interpretation of these terms. For example, “prudent use” refers to the overall goal of reducing usage, whereas “rational use” alludes to the administration of antimicrobials with the purpose of optimising treatment efficacy whilst minimising development of AMR [89].

There was also evidence of guidelines instructing variable strengths of obligation regarding specific recommendations. Levels of obligation sometimes overlapped and the strength of recommendation sometimes was not in agreement between guidelines. For example, some guidelines advised to “*reduce*”, “*discontinue*” or “*avoid*” prophylactic use of antimicrobials wherever possible, whilst others instructed to prohibit or reduce their “*routine*” use on farm. Wording of recommendations is of critical importance in communicating stakeholders’ intent of a recommendation. For example, when the use of antimicrobial sensitivity testing is strongly recommended in national guidelines, it is performed more regularly by veterinarians [32]. Hence, increasing the strength of recommendations across obligations could improve their practice.

In other cases, modal verbs were used inconsistently and interchangeably across guidelines. While some guidelines communicated a clear obligation not to use antimicrobials for prophylaxis:

“*Antibiotics must not be used for prophylactic purposes*.” [60]

others framed the use of prophylaxis as undesirable but permitted based on the veterinarians’ clinical discretion:

“*The use of antimicrobial products in the absence of clinical disease or specific pathogen infections and*, *in particular*, *administration to prevent disease should not be practised without a clear justification with respect to the health and welfare of the treated animals*.” [90]

Variation in modal verbs is likely the result of flexibility issued to guidelines to ensure they remain dynamic and appropriate to individual prescribing scenarios. Although antimicrobial use guidelines should form the basis of decision making, with the potential for different or additional approaches may be required in some clinical cases [56]. Guidelines that are inflexible leave insufficient room for clinical decisions to be tailored to individual circumstances [91], however variation in instruction may result in confusion between the voluntary and statutory natures of guidance, since different modal verbs elicit varying levels of obligation [80]. Whatever deontic terms stakeholders choose to employ, it is important that they are used consistently across guidelines [85].

In some cases, guidelines referred veterinarians to guidelines from other organisations, however, considering the degree of variation between guidelines, this may serve to weaken engagement and strengthen independent decisions.

Importantly, variation in guideline content may be the result of the shifting political and scientific landscape over the years, which will directly influence a change in the focus of guidelines. Guidelines are context-specific and time-specific; what is considered responsible use at one time-point may not be considered responsible at another [88]. Furthermore, the evolution of the concept of antimicrobial stewardship, coupled with the advancement of the evidence base underpinning recommendations, are also likely drivers of variation between guidelines [92].

#### Scope of content

The breadth and comprehensiveness of narrative guidelines produced by stakeholders varied considerably by the perceived target audience. Whilst all guidelines provided a rationale for responsible antimicrobial therapy, wide variation in stewardship messages was evidenced. Different social groups are likely to adopt different ways of framing the reality of responsible antimicrobial use, due to differences in roles and perspectives, which can lead to deep-seated differences in views about how recommendations should be governed [93]. Different organisational and political interests or agendas may also mean that guidelines will not always be synchronous between stakeholders. As such, the variety of guidelines studied is likely a product of stakeholders expressing the same concept differently in relation to their individual objectives.

Guidelines aimed at farmers were found to be more generic in nature and focused on the peripheral features of responsible use. Content in these guidelines was restricted to aspects considered most relevant for the audience. Noteworthy was the exclusion of information referring to diagnostic and sensitivity testing as well as choice of antimicrobial, dose and duration. This, of course, is expected given that antimicrobials are prescription-only medicines and can only be prescribed by a veterinarian [4], therefore farmers should not be guided to make these prescribing decisions. This contrasted veterinarian-directed guidelines which were generally more focused on the technical components, particularly the clinical and pharmacological indications for prescribing. Whilst farmers may be familiar with guidelines produced by milk and meat processors and certification bodies, findings from this study suggest that these may not always be the most comprehensive nor rigorous. However, developing comprehensive guidelines for farmer audiences could cause veterinarians to feel that their professional expertise is minimised by allowing farmers to decide on the use of antimicrobials. Given that farmers often use antimicrobials independently of veterinarian guidance, particularly when storing a range of antimicrobials on farm, the lack of comprehensive guidelines directed at farmers may be a deliberate effort to reduce farmer autonomy and improve the agency and ownership of the veterinarian legally responsible for antimicrobial use [94].

There was also an overwhelming lack of content relating to “under care” in farmer-directed guidance, compared to guidance aimed at veterinarians. This presents a significant gap in current guidelines and offers opportunity for stakeholders to educate farmers on the veterinarian Code of Professional Conduct and the responsibility of veterinarians to prescribe to animals under their care.

Some guidelines also appeared to be developed as public-facing messaging and were utilised as corporate communications or marketing strategies to engage with consumers. For example, retailers utilised antimicrobial policies to showcase their commitment to promoting the responsible use of antimicrobials within their food and supply chains by outlining the key principles informing antimicrobial use on supplier farms:

“*All antibiotics are used carefully*, *under strict protocols and only in controlled circumstances*… *Since 2014*, *Waitrose & Partners*, *in conjunction with its suppliers*, *have developed strategies and policies to reduce the total use of antibiotics across the supply chain*, *while keeping animal welfare at the forefront of all decision around veterinary medicine*.” [95]

### Recommendations for practice

Findings from this study reveal coexistent layers of tension within and between guidelines, highlighting significant implications for the clinical use of guidelines. Weaknesses identified in existing guidelines highlight considerable opportunities to enhance the transparency, reliability and applicability of guidelines, in order to direct behaviour aligned with the responsible use of antimicrobials in food-producing animals. With these concerns in mind, improvements could be achieved by incorporating the following recommendations.

#### Recommendation 1: Develop species-specific clinical disease guidelines to improve specificity of guidance

In human medicine, disease-specific clinical practice guidelines are a cornerstone of antimicrobial stewardship and a well-known tool for optimising antimicrobial use [70]. A major finding of this study is the lack of comprehensive disease-specific clinical guidelines relating to common diseases in cattle and sheep in the UK, which presents an important barrier to antimicrobial stewardship in food-producing animals. This was surprising considering the availability of specific clinical disease guidelines for small animals in the UK [96], and large animals in places like Australia [97]. Furthermore, in human medicine, the generation and publication of disease-specific guidelines in the UK is driven by the National Institute for Health and Care Excellence (NICE), with the aim to advise professionals with the National Health Service (NHS) on how to provide the highest standards of care.

Whist existing guidelines provide the basis for decision-making, it is difficult, if not impossible, to have a set of guidelines that can be applied universally [55], as recommendations for reducing inappropriate prescribing for one clinical scenario may not work for another [98]. Species-specific clinical disease guidelines and formularies are integral to efforts to reduce inappropriate prescribing by tailoring guidance to specific clinical scenarios, and should be prioritised.

#### Recommendation 2: Expand guidelines to include preventative measures

Infection control and disease prevention play an important role in veterinary antimicrobial stewardship [48,49]. In this study, some guidelines advocated disease prevention measures as the cornerstone of antimicrobial stewardship initiatives to mitigate antimicrobial use, yet disease prevention recommendations were not behaviourally specific nor provided sufficient in detail for measures to be practically implemented. This is likely to be a result of the lack of disease-specific guidelines, meaning that surface-level recommendations have only been considered appropriate.

For species-specific clinical disease guidelines to be stand-alone tools, greater attention should be paid to the role of preventive measures in responsible antimicrobial use. Inclusion of preventive measures within these guidelines could help reinforce the importance of disease prevention within antimicrobial stewardship strategies and substantiate veterinary advice to farmers [29]. This could be facilitated by building guidance around the ‘plan, prevent, protect’ framework [99] using the available evidence base to support recommended measures.

#### Recommendation 3: Provide definitions to resolve vagueness and yield consistent interpretation and intent

Central to antimicrobial stewardship is the need for clear guidelines on antimicrobial usage [100]. However, the vagueness and ambiguity of guidance reported in this study are likely contributors to the variation seen in prescribing practices by veterinarians. Vaguely defined or undefined terminology and guidance requires the veterinarian to “fill in the blanks”.

It is important that terminology is standardised, with clear specification of obligation, with well-defined and prescriptive guidance, to resolve vagueness and reduce opportunity for alternative interpretation [68]. Providing behaviourally specific, practical definitions of terms leads to stronger intentions to implement recommended practices [72,101]. This could include defining routine prophylaxis, “under care” and the conditions for which strategic treatments are considered to align with responsible prescribing practices. Providing real-life examples of prescribing challenges and scenarios should be considered when developing guidelines.

#### Recommendation 4: Develop guidelines within practice to build collaboration and ownership

Developing guidelines at practice-level can improve the agreement of prescribing behaviours between veterinarians within a practice. Firstly, involvement of veterinarians in the development of their own guidelines can bolster ownership of and subsequent compliance to guidelines [102]. Working towards a mutual goal could create a sense of unity, which would subsequently promote positive feelings towards the collaboration in general [103]. This is important considering conformity to prescribing guidelines can be thwarted by differences in opinion or resentment towards recommendations [104], particularly when guidelines are regarded as threatening professional autonomy [105]. Implementation of practice-level guidelines could be just the trigger for mindset and behavioural change needed [106].

Secondly, development of in-practice guidelines can reduce unnecessary variation in prescribing practices. Veterinarians can be placed in difficult situations when animal owners have previously seen colleagues set a precedent by prescribing antimicrobials in conflict with guidelines [107]. Farmers have been reported to perceive variation in the advice given by veterinarians regarding antimicrobial use [44]. This lack of consistency in stewardship messaging between veterinarians may erode trust that farmers place in veterinarians, reducing their motivation to change antimicrobial usage [44]. Additionally, facilitating uniform prescribing practices between veterinary practices–through shared adoption of in-practice guidelines–could mitigate instances where farmers seek antimicrobials from alternative veterinary practices with known differences in prescribing behaviours.

Thirdly, collaboration within a practice may also help support newly qualified veterinarians, where practice prescribing guidelines may provide the additional support these veterinarians seek in order to act independently of farmers’ demands [10]. Practice protocols may build confidence and guide more junior veterinarians to make more independent decisions [12]. Ensuring veterinarians are prescribing in accordance with set practice guidelines also ensures that newly qualified veterinarians are exposed to consistent prescribing behaviours within practice, as these veterinarians may limit their actions to fit in with their colleagues’ habits [24].

#### Recommendation 5: Unite stakeholders to align priorities and messaging

Encouraging stakeholders to join forces and cultivate collaboration is instrumental in aligning perspectives and priorities necessary for driving a consistent message of responsible use. Fostering active working between stakeholders will thwart the application of contradictory or inconsistent recommendations, deontic terms and levels of obligation. In doing so, streamlining the composition of guidelines and broadening the representation of voices may increase the validity and acceptability of resultant guidelines [108] in addition to reducing the ‘noise’ associated with a large number of often overlapping guidelines, as analysed in this study. The production of a national responsible use guideline, facilitated by a range of stakeholders from farm to fork and supported by veterinarians, could complement the development of species-specific clinical disease guidelines.

### Methodological considerations

This is the first study to explore and evaluate antimicrobial use guidelines relating to cattle and sheep in the UK from a qualitative perspective. This was a purposive sample of 128 veterinary guidelines on antimicrobial use pertaining to cattle and sheep in the UK, and thus, the findings from this study cannot claim to be representative of all veterinary guidelines for antimicrobial use. Limitations of this study include possible selection bias due to sourcing only publicly available, online documents, although a highly varied dataset of material was sourced. Whilst an established, systematic methodology was undertaken, alternative approaches to analysis exist; future studies are therefore recommended to increase the scope of analysis and interpretation of findings. Further research is also required to evaluate the impact of responsible use guidelines in terms of feasibility, usability, prescriber adherence and impact on total and specific antimicrobial usage.

## Conclusions

Guidelines for the responsible use of antimicrobials play an important role in directing veterinarian behaviour in order to reduce the use of antimicrobials and minimise the development of AMR in veterinary medicine. National and international guidelines have been developed to address antimicrobial prescribing in livestock species. This study aimed to explore and evaluate the current landscape of antimicrobial use guidelines pertinent to cattle and sheep in the UK. Findings from rigorous qualitative assessment revealed four crosscutting themes relating to the roles and responsibilities of the veterinarian and the realities of the prescribing paradigm, in addition to questions concerning the specificity within and congruence between guidelines. Subsequent recommendations for practice have considerable potential to shape the future development of antimicrobial use guidelines. In particular, transforming existing general principles of responsible antimicrobial use into a set of species-specific and disease-specific guidelines could prove valuable within the ruminant sector. Whilst making guidelines fit for purpose will not be the magic bullet for antimicrobial prescribing and AMR, this action instead offers a solution as part of the multidimensional antimicrobial stewardship interventions that seek to improve responsible antimicrobial use.

## Supporting information

S1 Table Full list of all guidelines on antimicrobial use (n = 128) reviewed in the study. (PDF)
