# Supplementary material for: Validation, visibility, vagueness and variation: A qualitative assessment of existing veterinary guidelines for antimicrobial use in cattle and sheep in the UK
Source: PLoS One. 2023 Nov 30;18(11):e0294733. doi: 10.1371/journal.pone.0294733 (PMC10688698; doi:10.1371/journal.pone.0294733)
Supplement: S1 Table — (PDF) [file pone.0294733.s001.pdf]

Supporting Information

S1 Table. Full list of all guidelines on antimicrobial use (n = 128) reviewed in the study.

	Stakeholder	Reference
**Milk & meat processors**		
1	**ABP Food Group**	1. ABP Food Group. 2023. Livestock Policy. [Online]. Available from: https://abpfoodgroup.com/agriculture/livestock-policy/ [Accessed 11th October 2023].
2	**Arlagården®**	2. Arlagården®. 2017. Arlagården standards. [Online]. Available from: https://www.arlafoods.co.uk/4a3b63/globalassets/about-arla/our-responsibility/standards---updated-from-1-january-2017.pdf [Accessed 11th October 2023].
3		3. Arlagården®. 2021. Farm Management Programme. [Online]. Available from: https://www.arla.com/4a54d6/globalassets/arla-global/arlagaarden/arlagardenrcatalogue.pdf [Accessed 11th October 2023].
4	**Arla Foods**	4. Arla Foods. 2023. Animal Welfare. [Online]. Available from: https://news.arlafoods.co.uk/sustainable/animal-welfare [Accessed 11th October 2023].
5	**Cranswick**	5. Cranswick. 2018. Group Antimicrobial Resistance Policy. [Online]. Available from: https://cranswick.plc.uk/sites/default/files/Group%20Antimicrobial%20Resistance%20Policy.pdf [Accessed 11th October 2023].
6		6. Cranswick. 2020. Animal Health & Welfare 2019. [Online]. Available from: https://cranswick.plc.uk/sites/default/files/11980_CWK_FC_Animal%20Welfare%20Brochure_v7_AW.pdf [Accessed 11th October 2023].
7	**Milk & More**	7. Milk & More. Not Dated. Our Animal Welfare Policy. [Online]. Available from: https://www.milkandmore.co.uk/our-animal-welfare-policy [Accessed 11th October 2023].
8	**Nestlé**	8. Nestlé. 2021. Nestlé Farm Animal Welfare Q&A. Management Commitment and Policy. [Online]. Available from: https://www.nestle.com/sites/default/files/2019-08/nestle-farm-animal-welfare-qa.pdf [Accessed 11th October 2023].
9		9. Nestlé. 2018. Nestlé Responsible Sourcing Standard. [Online]. Available from: https://www.nestle.com/sites/default/files/asset-library/documents/library/documents/suppliers/nestle-responsible-sourcing-standard-english.pdf [Accessed 11th October 2023].
10	**First Milk**	10. First Milk. 2022. Executive Summary Review of the year 2021/2022. [Online]. Available from: https://www.firstmilk.co.uk/app/media/First-Milk-AR2022-EMAIL.pdf [Accessed 11th October 2023].
11	**Foyle Food Group**	11. Foyle Food Group. 2021. Animal Welfare Policy. [Online]. Available from: http://www.foylefoodgroup.com/wp-content/uploads/2021/08/Foyle-Animal-Welfare-Policy.pdf [Accessed 11th October 2023].
12		12. Foyle Food Group. 2021. Our Welfare Promise. Animal Welfare at Foyle Food Group. [Online]. Available from: https://www.foylefoodgroup.com/wp-content/uploads/2021/08/Foyle-Food-Group-Animal-Welfare.pdf [Accessed 11th October 2023].
13	**Kepak**	13. Kepak. 2017. Animal Welfare Policy. [Online]. Available from: https://www.kepak.com/assets/kepak-animal-welfare-policy.pdf [Accessed 11th October 2023].

14		14. Kepak. 2017. AMR Statement. [Online]. Available from: https://www.kepak.com/farming/amr/ [Accessed 11th October 2023].
15	**Lactalis UK and Ireland**	15. Kepak. 2022. Lactalis Group Animal Welfare Policy. [Online]. Available from: https://www.lactalis.com/wp-content/uploads/2023/05/Animal-welfare-Caring-for-animals-all-along-our-value-chain.pdf [Accessed 11th October 2023].
16	**Meadow Foods**	16. Meadow Foods. 2022. Animal Health and Welfare Policy. [Online]. Available from: https://meadowfoods.co.uk/wp-content/uploads/2022/10/Meadow_Animal_welfare_policy_v5.pdf [Accessed 11th October 2023].
17	**Premier Foods**	17. Premier Foods. 2022. Premier Foods Animal Welfare Policy. [Online]. Available from: https://www.premierfoods.co.uk/CorporateSite/media/documents/responsibility/animal_welfare/Animal-Welfare-Policy-Mar-22.pdf [Accessed 11th October 2023].
18	**Saputo**	18. Saputo. Not Dated. Position Paper on Animal Health and Welfare Issues. [Online]. Available from: https://saputo.com/en/our-promise/responsible-sourcing/animal-welfare?msclkid=3e441bb0c18e11ec9444a147b4e18c10 [Accessed 11th October 2023].
19	**Pilgrim's UK**	19. Pilgrim's UK. 2022. Animal Welfare Policy. [Online]. Available from: https://www.pilgrimsuk.com/app/uploads/2022/12/Pilgrims-UK-Animal-Welfare-Policy-Dec-2022.pdf [Accessed 11th October 2023].
20	**Hilton Food Group Plc**	20. Hilton Food Group Plc. 2020. Hilton Food Group Animal Welfare Policy. [Online]. Available from: https://www.hiltonfoods.com/media/cxmflkkz/hfg-animal-welfare-statement-for-calender-year-2020.pdf [Accessed 11th October 2023].
**Retailer & restaurants**		
1	**ALDI UK**	21. ALDI UK. 2020. Animal Welfare Policies and Performance. [Online]. Available from: https://cdn.aldi-digital.co.uk/qtnOt8Js@VBkpbdasvro0UdWKWs.pdf [Accessed 11th October 2023].
2	**ASDA**	22. ASDA. 2018. Reducing antibiotics in food production. [Online]. Available from: http://s7d2.scene7.com/is/content/asdagroceries/Asda.com/7.%20Sites/Environment/ASDA-Antibiotics-report-2018_V7.pdf [Accessed 11th October 2023].
3	**Co-op**	23. Co-op. Not Dated. Co-op Animal Welfare Standards & Performance & Co-op Antibiotics Policy. [Online]. Available from: https://assets.ctfassets.net/bffxiku554r1/2l3meYk2jLxPpKWTARwCWR/628e67df6e119d2cd8ae7012578d8ca6/Co-op_Animal_Welfare_Standards__Performance_and_Co-op_Antibiotic_Policy_v4.pdf [Accessed 11th October 2023].
4	**Greggs**	24. Greggs. 2023. Farm Animal Welfare Strategy 2023. [Online]. Available from: https://a.storyblok.com/f/162306/x/331e206949/19540-greggs-farm-animal-welfare-strategy-2023_a4_30-03-23.pdf [Accessed 11th October 2023].
5	**Iceland**	25. Iceland. 2023. Animal Welfare. [Online]. Available from: https://sustainability.iceland.co.uk/our-planet/sustainable-sourcing/animal-welfare/ [Accessed 11th October 2023].
6	**John Lewis Partnership**	26. John Lewis Partnership. 2023. Our animal welfare policy and livestock KPIs. [Online]. Available from: https://www.johnlewispartnership.co.uk/content/dam/cws/pdfs/Juniper/ethics-and-sustainability/Our-animal-welfare-policy-and-livestock-KPIs-January%202023.pdf [Accessed 11th October 2023].

7	**Marks and Spencer**	27. Marks and Spencer. 2021. Antibiotic Usage Policy in M&S Sourced Livestock and Aquaculture. [Online]. Available from: https://corporate.marksandspencer.com/sites/marksandspencer/files/marks-spencer/sustainability/our-products/marks-and-spencer-antibiotic-policy-2021-new.pdf [Accessed 11th October 2023].
8	**McDonald's**	28. McDonald's. Not Dated. Antibiotic Use Policy for Beef and Dairy Beef. [Online]. Available from: https://corporate.mcdonalds.com/content/dam/sites/corp/nfl/pdf/McDonalds_Beef_Antibiotics_Policy.pdf [Accessed 11th October 2023].
9		29. McDonald's. 2017. McDonald's Global Vision for Antibiotic Stewardship in Food Animals ("VAS"). "Preserving Antibiotic effectiveness in the future through ethical practices today". [Online]. Available from: https://corporate.mcdonalds.com/content/dam/sites/corp/nfl/pdf/McDonalds-Global-Vision-for-Antimicrobial-Stewardship-in-Food.pdf [Accessed 11th October 2023].
10		30. McDonald's. 2022. Responsible Antibiotic Use. Available from: https://corporate.mcdonalds.com/corpmcd/our-purpose-and-impact/food-quality-and-sourcing/responsible-antibiotic-use.html [Accessed 11th October 2023].
11		31. McDonald's. 2022. McDonald's Antibiotic Policy for our Beef Supply Chain. [Online]. Available from: https://corporate.mcdonalds.com/content/dam/sites/corp/nfl/pdf/McDonalds_Beef_and_Dairy%20_Antibiotic_Policy.pdf [Accessed 11th October 2023].
12	**Morrisons Farming**	32. Morrisons Farming. 2023. Antibiotic Use. [Online]. Available from: https://www.morrisons-farming.com/how-we-work/antibiotic-use/ [Accessed 11th October 2023].
13		33. Morrisons Farming. 2023. Antibiotics Policy. [Online]. Available from: https://www.morrisons-corporate.com/morrisons-sustainability/policies/antibiotics-policy/ [Accessed 11th October 2023].
14	**Sainsbury's**	34. Sainsbury's. 2017. Animal Health & Welfare Report. [Online]. Available from: https://www.about.sainsburys.co.uk/~/media/Files/S/Sainsburys/pdf-downloads/animal-health-and-welfare.pdf [Accessed 11th October 2023].
15		35. Sainsbury's. 2019. Antibiotic Resistance: Keeping Our Animals Healthy. [Online]. Available from: https://www.about.sainsburys.co.uk/~/media/Files/S/Sainsburys/CRS%20Policies%20and%20Reports/Antibiotic-Resistance-Report-2019.pdf [Accessed 11th October 2023].
16		36. Sainsbury's. 2022. Sainsbury's Antibiotic Stewardship Report 2022. [Online]. Available from: https://www.about.sainsburys.co.uk/~/media/Files/S/Sainsburys/CRS%20Policies%20and%20Reports/Antibiotic%20Report_2022.pdf [Accessed 11th October 2023].
17	**Tesco**	37. Tesco. 2021. Antibiotics. [Online]. Available from: https://www.tescoplc.com/media/757859/antibiotic-update-202021.pdf [Accessed 11th October 2023].
18	**Waitrose**	38. Waitrose Farming Partnership. 2017. Animal Welfare at Waitrose. [Online]. Available from: https://www.waitrose.com/content/dam/waitrose/Inspiration/Waitrose%20Way/Animal%20welfare/FINAL%20BBFAW%20narrative%202017.pdf [Accessed 11th October 2023].
**Farm certification bodies**		
1	**Red Tractor**	39. Red Tractor. 2018. Responsible Use of Antibiotics on Red Tractor Dairy Farms. [Online]. Available from: https://redtractorassurance.org.uk/wp-content/uploads/2021/08/Responsible-use-of-antibiotics-on-Red-Tractor-Dairy-Farms.pdf [Accessed 11th October 2023].
2	**RSPCA**	40. RSPCA. 2020. RSPCA welfare standards for Beef cattle. [Online]. Available from: https://science.rspca.org.uk/documents/1494935/9042554/RSPCA+welfare+Beef+Cattle+February+2020.pdf/e29acbdc-f39f-2852-831b-48b6f296d4b6?t=1583410390866 [Accessed 11th October 2023].

3		41. RSPCA. 2020. RSPCA welfare standards for Sheep. [Online]. Available from: https://www.rspca.org.uk/documents/1494935/9042554/RSPCA+welfare+standards+for+sheep+%28PDF+10.3MB%29.pdf/e91f2d1e-4a04-30cd-5ed8-8f55da4513c6?t=1594889570996 [Accessed 11th October 2023].
4		42. RSPCA. 2021. RSPCA welfare standards for Dairy cattle. [Online]. Available from: https://science.rspca.org.uk/documents/1494935/9042554/RSPCA+Dairy+Standards.pdf/bb1a2971-8018-b325-8dcc-d6b590fcd955?t=1625822966824 [Accessed 11th October 2023].
5		43. RSPCA. 2022. Antimicrobial resistance (AMR) and farm animal welfare. [Online]. Available from: https://www.rspca.org.uk/documents/1494939/7712578/Antimicrobial+resistance.pdf/fda52d57-2b5a-f523-9a89-464b552a7355?t=1553271778548 [Accessed 11th October 2023].
6	**Soil Association**	44. Soil Association. 2021. Soil Association Standards Farming and growing. [Online]. Available from: https://www.soilassociation.org/media/15931/farming-and-growing-standards.pdf [Accessed 11th October 2023].
**Levy boards, associations & networks**		
1	**Agriculture and Horticulture Development Board (AHDB)**	45. AHDB. 2017. Using medicines correctly for Better Returns. [Online]. Available from: https://media.ahdb.org.uk/media/Default/Imported%20Publication%20Docs/Using%20medicines%20correctly%20for%20better%20returns.pdf [Accessed 11th October 2023].
2		46. AHDB. 2019. Antibiotic resistance. Using antibiotics responsibly. [Online]. Available from: https://projectblue.blob.core.windows.net/media/Default/Beef%20&%20Lamb/AMRLeaflet2019_190207_WEB-1.pdf [Accessed 11th October 2023].
3		47. AHDB. 2020. Using medicines responsibly. [Online]. Available from: https://projectblue.blob.core.windows.net/media/Default/Beef%20&%20Lamb/Using%20Medicines3784_200818_WEB.pdf [Accessed 11th October 2023].
4	**EuroDairy**	48. EuroDairy. 2018. Practical strategies to reduce antimicrobial use in dairy farming. [Online]. Available from: https://eurodairy.co.uk/media/1928/d58-practical-strategies-to-reduce-antimicrobials.pdf [Accessed 11th October 2023].
5	**Hybu Cig Cymru / Meat Promotion Wales**	49. Hybu Cig Cymru / Meat Promotion Wales. 2012. Herd health: Maximising your herd's potential through improved health. [Online]. Available from: https://meatpromotion.wales/images/resources/Herd_health_-_Maximising_your_herd%E2%80%99s_potential_through_improved_health.pdf [Accessed 11th October 2023].
6	**National Sheep Association (NSA)**	50. National Sheep Association. 2019. NSA policy position on responsible antimicrobial use in sheep. [Online]. Available from: https://www.nationalsheep.org.uk/workspace/pdfs/nsa-policy-position-on-responsible-antimicrobial-use-in-sheep.pdf [Accessed 11th October 2023].
7	**Quality Meat Scotland (QMS)**	51. Quality Meat Scotland. 2022. Cattle & Sheep Standards 2022. [Online]. Available from: https://s3.eu-west-2.amazonaws.com/quality-meat-scotland/documents/Standards/qms_cattle_and_sheep_standards_2022_spreads_set-up.pdf [Accessed 11th October 2023].
8	**Livestock & Meat Commission**	52. Livestock & Meat Commission. 2021. Practical Steps to Reduce Antimicrobial Usage on Your Farm. [Online]. Available from: https://www.lmcni.com/site/wp-content/uploads/2021/08/28082021.pdf [Accessed 11th October 2023].
**Farming unions**		
1	**NFU**	53. NFU. Not Dated. NFU responds to independent review on AMR. [Online]. Available from: https://www.nfuonline.com/archive?treeid=56432 [Accessed 11th October 2023].

Veterinary, animal health & medicine organisations		
1	**AnimalhealthEurope**	54. Animal Health Europe. Not Dated. Antibiotic resistance. [Online]. Available from: https://animalhealtheurope.eu/focus-areas/antibiotic-resistance/ [Accessed 11th October 2023].
2		55. AnimalhealthEurope. 2021. Impressive decrease in veterinary antibiotic use shows good uptake of Responsible Use principles in Europe. [Online]. Available from: https://animalhealtheurope.eu/wp-content/uploads/2022/01/ESVAC2021_AnimalhealthEurope_PressStatement231121.pdf [Accessed 11th October 2023].
3		56. AnimalhealthEurope. 2022. The vet's toolbox will always require antibiotics. [Online]. Available from: https://animalhealtheurope.eu/wp-content/uploads/2022/01/AnimalhealthEuropeComm_WhyVetsNeedAntibiotics_FIN.pdf [Accessed 11th October 2023].
4	**British Cattle Veterinary Association (BCVA)**	57. BCVA. 2016. British Cattle Veterinary Association promotes the responsible use of medicines. [Online]. Available from: https://www.bcva.org.uk/system/files/resources/BCVA%20AMR%20Statement%20December%202016.pdf [Accessed 11th October 2023].
5	**British Veterinary Association (BVA)**	58. BVA. 2013. Antibiotics – your role as a farmer. [Online]. Available from: https://www.southwalesfarmvets.co.uk/documents/your-role.pdf [Accessed 11th October 2023].
6		59. BVA. 2019. BVA position on the responsible use of antimicrobials in food producing animals. [Online]. Available from: https://www.bva.co.uk/media/1161/bva-policy-position-on-the-responsible-use-of-antimicrobials-in-food-producing-animals-1.pdf [Accessed 11th October 2023].
7		60. BVA. 2019. Responsible use of antimicrobials in veterinary practice: The 7-point plan. [Online]. Available from: https://www.vettimes.co.uk/app/uploads/2019/05/20190508-BVA-AMR-poster-2019-v4.pdf [Accessed 11th October 2023].
8	**European Platform for the Responsible Use of Medicines in Animals (EPRUMA)**	61. EPRUMA. 2015. Responsible Use of medicines in animals: what does this mean? [Online]. Available from: http://www.sustainable-agriculture.org/wp-content/uploads/2015/04/EPRUMAfactsheetfin-20150421.pdf [Accessed 11th October 2023].
9		62. EPRUMA. 2015. EPRUMA best-practice framework for the use of antibiotics in food-producing animals. [Online]. Available from: https://epruma.eu/wp-content/uploads/2019/04/EPRUMAABNextlevel-Englis.pdf [Accessed 11th October 2023].
10		63. EPRUMA. 2021. 10 Myth Busting Facts about Antibiotics and Animals. [Online]. Available from: https://fve.org/cms/wp-content/uploads/Myths-and-facts-EPRUMA-FINAL-300821.pdf [Accessed 11th October 2023].
11	**Federation of Veterinarians of Europe (FVE)**	64. FVE. 2014. Using antimicrobials responsibly. Advice for Doctors, Dentists and Veterinarians. [Online]. Available from: https://fve.org/cms/wp-content/uploads/FVE_sheet_vet_doctor_dentist_EN_november_2014_web.pdf [Accessed 11th October 2023].
12		65. FVE. 2020. Joint AVMA-FVE-CVMA statement on responsible and judicious use of antimicrobials. [Online]. Available from: https://fve.org/cms/wp-content/uploads/AVMA_FVE_CVMA_Antimicrobial_JointStatement_17April2020.docx.pdf [Accessed 11th October 2023].
13		66. NADIS. Not Dated. Antibacterial Use in Cattle. [Online]. Available from: https://www.nadis.org.uk/disease-a-z/cattle/antibacterial-use-in-cattle/ [Accessed 11th October 2023].

14	**National Animal Disease Information Service (NADIS)**	67. NADIS. Not Dated. Sheep medicines. [Online]. Available from: https://www.nadis.org.uk/disease-a-z/sheep/sheep-medicines/ [Accessed 11th October 2023].
15	**National Office of Animal Health (NOAH)**	68. NOAH. 2016. Responsible Use of Antibiotics. [Online]. Available from: https://www.noah.co.uk/wp-content/uploads/2022/06/NOAH-BD-Responsible-Use-of-Antibiotics-10-05-16.pdf [Accessed 11th October 2023].
16		69. NOAH. 2016. Antibiotics for Animal Health and Welfare: An Overview. [Online]. Available from: https://www.noah.co.uk/wp-content/uploads/2022/06/NOAH-BD-Antibiotics-for-Animal-Health-Welfare-10-05-1.pdf [Accessed 11th October 2023].
17		70. NOAH. 2017. Animal health sector commitments and actions on antibiotic use. [Online]. Available from: https://www.noah.co.uk/wp-content/uploads/2017/10/NOAH-HFA-Commitments-Project-Oct-17-v2.pdf [Accessed 11th October 2023].
18		71. NOAH. 2019. Code of Practice for the Promotion of Animal Medicines. [Online]. Available from: https://www.noah.co.uk/wp-content/uploads/2021/04/Code-of-Practice-Booklet-29-effective-December-2019-AMENDED.pdf [Accessed 11th October 2023].
19		72. NOAH. 2019. Our Vision for UK Animal Health and Welfare. [Online]. Available from: https://www.noah.co.uk/wp-content/uploads/2022/06/NOAH-Vision-Paper-8pp-A4-Sep-19-vf.pdf [Accessed 11th October 2023].
20		73. NOAH. 2021. NOAH Technical Briefing: Categorisation of Antibiotics and Updated Guidance to Support Responsible Use and UK Animal Health and Welfare. [Online]. Available from: https://www.noah.co.uk/wp-content/uploads/2021/12/2021-12-07-NOAH-briefing-on-updated-EMA-Categorisation-Guidelines-FINAL.pdf [Accessed 11th October 2023].
21		74. NOAH. 2021. NOAH Briefing: Responsible Use of Antibiotics in Cattle: Best practice and an evidenced-based approach to macrolide policy and prescribing. [Online]. Available from: https://www.noah.co.uk/wp-content/uploads/2022/08/2021-12-07-Responsible-Use-of-Antibiotics-in-Cattle-NOAH-briefing-FINAL.pdf [Accessed 11th October 2023].
22	**Responsible Use of Medicines in Agriculture Alliance (RUMA)**	75. RUMA. 2022. Information Note on Antibiotic Resistance and the Responsible Use of Antibiotics in Farm Animals. [Online]. Available from: https://ruma.org.uk/wp-content/uploads/2022/07/Responsible-Use-of-Antibiotics-in-Farm-Animals-General-Guidance.pdf [Accessed 11th October 2023].
23		76. RUMA. 2022. RUMA Position Statement on the Preventive Use of Antibiotics in Farm Animals. [Online]. Available from: https://ruma.org.uk/wp-content/uploads/2022/07/The-Preventive-Use-of-Antibiotics-in-Farm-Animals-Prophylaxis.pdf [Accessed 11th October 2023].
24		77. RUMA. 2022. Completion of antibiotic treatment courses. [Online]. Available from: https://ruma.org.uk/wp-content/uploads/2022/07/Completion-of-antibiotic-treatment-courses.pdf [Accessed 11th October 2023].
25		78. RUMA. 2022. Risk categorisation for antibiotics. [Online]. Available from: https://ruma.org.uk/wp-content/uploads/2022/07/Risk-categorisation-for-antibiotics.pdf [Accessed 11th October 2023].
26		79. RUMA. 2022. Guidelines for Responsible use of antimicrobials in cattle production. [Online]. Available from: https://www.ruma.org.uk/wp-content/uploads/2023/06/RUMA_antimicrobial_long_cattle_-2022-revisions-Final-.pdf [Accessed 11th October 2023].
27		80. RUMA. 2022. Guidelines on Responsible Use of Antimicrobials in Dry Cow Strategies. [Online]. Available from: https://ruma.org.uk/wp-content/uploads/2022/08/Guidelines-on-Responsible-Use-of-Antimicrobials-in-Dry-Cow-Strategies.pdf [Accessed 11th October 2023].

28		81. RUMA. 2022. About Antibiotic Resistance. [Online]. Available from: https://www.ruma.org.uk/about-antibiotic-resistance/ [Accessed 11th October 2023].
29	**Royal College of Veterinary Surgeons (RCVS)**	82. RCVS. 2016. Code of Professional Conduct for Veterinary Surgeons and Supporting Guidance. [Online]. Available from: https://www.rcvs.org.uk/setting-standards/advice-and-guidance/code-of-professional-conduct-for-veterinary-surgeons/pdf/ [Accessed 11th October 2023].
30		83. RCVS. 2023. Veterinary medicines. [Online]. Available from: https://www.rcvs.org.uk/setting-standards/advice-and-guidance/code-of-professional-conduct-for-veterinary-surgeons/supporting-guidance/veterinary-medicines/ [Accessed 11th October 2023].
31	**Scotland's Healthy Animals**	84. Scotland's Healthy Animals. Not Dated. Create an Antimicrobial Stewardship Policy or Programme (ASP). [Online]. Available from: https://www.scotlandshealthyanimals.scot/keep-antibiotics-working/create-an-antimicrobial-stewardship-policy-or-programme-asp/create-an-antimicrobial-stewardship-policy-or-programme-asp/ [Accessed 11th October 2023].
32	**Sheep Antibiotic Guardian Group (SHAWG)**	85. SHAWG. 2019. Industry Guidance Document for Veterinary Surgeons and Farmers on Responsible Use of Antibiotics in Sheep. [Online]. Available from: https://projectblue.blob.core.windows.net/media/Default/Beef%20&%20Lamb/SHAWG/Responsible-AMU-guidance-for-sheep_Version-1.0_June-2019.pdf [Accessed 11th October 2023].
33	**Sheep Veterinary Society (SVS)**	86. SVS. Not Dated. SVS Antibiotics Policy. [Online]. Available from: https://sheepvetsoc.org.uk/knowledge-hub/svs-antibiotics-policy/ [Accessed 11th October 2023].
34		87. SVS. 2017. Responsible Use of Antimicrobials Good Practice Guidelines. [Online]. Available from: https://sheepvetsoc.org.uk/wp-content/uploads/2021/04/SVS-Good-Practice-Guidelines.pdf [Accessed 11th October 2023].
35		88. SVS. 2022. Sheep Veterinary Society /Sheep Antibiotic Guardian Group Document written to advise vets on control of watery mouth in neonatal lambs in the face of cessation of production of Spectam Scour Halt. [Online]. Available from: https://sheepvetsoc.org.uk/wp-content/uploads/2022/02/Updated-Spectam-guidance-SVS-11March22_final.pdf [Accessed 11th October 2023].
36	**Teagasc**	89. Teagasc. Not Dated. Critically Important Antibiotics (CIAs). [Online]. Available from: https://www.teagasc.ie/animals/amr/critically-important-antibiotics-cias/ [Accessed 11th October 2023].
37		90. Teagasc. Not Dated. Code of Good Practice Regarding the Responsible Use of Antimicrobials on Dairy Farms. [Online]. Available from: https://www.teagasc.ie/media/website/animals/amr/Code-of-Good-Practice-for-Responsible-Use-of-AMs-on-Dairy-Farms.pdf [Accessed 11th October 2023].
38		91. Teagasc. 2018. Prudent Use of Antibiotics. [Online]. Available from: https://www.teagasc.ie/animals/amr/prudent-use/ [Accessed 11th October 2023].
39	**World Veterinary Association (WVA)**	92. WVA. 2018. World Veterinary Association Policy on Responsible Use of Antimicrobials. [Online]. Available from: https://worldvet.org/uploads/docs/wva_revision_2_-_position_on_the_use_of_antimicrobials_2018.pdf [Accessed 11th October 2023].
40		93. WVA. 2018. Pharmaceutical Stewardship" – A Strategic Priority for the World Veterinary Association. [Online]. Available from: https://worldvet.org/policies/pharmaceutical-stewardship-a-strategic-priority-for-the-world-veterinary-association/ [Accessed 11th October 2023].

41		94. WVA. 2021. World Veterinary Association Position on the Role of Veterinarians in Food Safety. [Online]. Available from: https://worldvet.org/wp-content/uploads/2022/03/position_on_the_role_of_veterinarians_in_food_safety.pdf [Accessed 11th October 2023].
**Farm and business support**		
1	**FAI Farms**	95. FAI Farms. 2018. FAI Presents a 3Rs Approach to Antimicrobial Stewardship in Livestock Supply Chains. [Online]. Available from: https://www.faifarms.com/portfolio-item/fai-presents-a-3rs-approach-to-antimicrobial-stewardship-in-livestock-supply-chains/ [Accessed 11th October 2023].
**Government organisation & governing bodies**		
1	**Department of Agriculture, Environment and Rural Affairs (DAERA)**	96. DAERA. 2014. Responsible use of Antimicrobials in Livestock. [Online]. Available from: https://www.daera-ni.gov.uk/sites/default/files/publications/dard/responsible-use-of-antimicrobials-in-livestock.pdf [Accessed 11th October 2023].
2		97. DAERA. 2021. Handle Antimicrobials with Care. [Online]. Available from: https://www.daera-ni.gov.uk/sites/default/files/publications/daera/21.22.082%20Antimicrobials%20-AMR%20A4%20Leaflet%20Final.PDF [Accessed 11th October 2023].
3	**Department of Agriculture, Food and the Marine**	98. Department of Agriculture, Food and the Marine. 2020. Policy on Highest Priority Critically Important Antimicrobials. [Online]. Available from: https://www.gov.ie/pdf/?file=https://assets.gov.ie/94006/71a8d1e0-bfe4-40a2-b15a-b7c7e45278f5.pdf#page=1 [Accessed 11th October 2023].
4	**European Commission**	99. European Commission. 2015. Guidelines for the prudent use of antimicrobials in veterinary medicine. [Online]. Available from: https://health.ec.europa.eu/system/files/2016-11/2015_prudent_use_guidelines_en_0.pdf [Accessed 11th October 2023].
5		100. European Commission. 2020. A European One Health Action Plan against Antimicrobial Resistance (AMR). [Online]. Available from: https://health.ec.europa.eu/system/files/2020-01/amr_2017_action-plan_0.pdf [Accessed 11th October 2023].
6	**Houses of Parliament: Parliamentary Office of Science and Technology**	101. Houses of Parliament. 2018. Reducing UK Antibiotic Use in Animals. [Online]. Available from: https://researchbriefings.files.parliament.uk/documents/POST-PN-0588/POST-PN-0588.pdf [Accessed 11th October 2023].
7	**Department of Health and Social Care**	102. Department of Health and Social Care. 2019. Contained and controlled: The UK's 20-year vision for antimicrobial resistance. [Online]. Available from: https://assets.publishing.service.gov.uk/media/5c48896a40f0b616fe901e91/uk-20-year-vision-for-antimicrobial-resistance.pdf [Accessed 11th October 2023].
8		103. Department of Health and Social Care. 2019. Tackling antimicrobial resistance 2019 to 2024: the UK's 5-year national action plan. [Online]. Available from: https://assets.publishing.service.gov.uk/media/6261392d8fa8f523bf22ab9e/UK_AMR_5_year_national_action_plan.pdf [Accessed 11th October 2023].
9	**House of Commons Library**	104. House of Commons Library. 2023. The use of antibiotics on healthy farm animals and antimicrobial resistance. [Online]. Available from: https://researchbriefings.files.parliament.uk/documents/CDP-2023-0012/CDP-2023-0012.pdf [Accessed 11th October 2023].
10	**Veterinary Medicines Directorate (VMD)**	105. VMD. Not Dated. Antimicrobials. [Online]. Available from: https://assets.publishing.service.gov.uk/media/5a7f070740f0b62305b84ab4/_253645_Leaflet_004_A_-_Antimicrobials.pdf [Accessed 11th October 2023].

11		106. VMD. 2014. Code of Practice on the responsible use of animal medicines on the farm. [Online]. Available from: https://www.gov.uk/government/publications/responsible-use-of-animal-medicines-on-the-farm/code-of-practice-on-the-responsible-use-of-animal-medicines-on-the-farm [Accessed 11th October 2023].
12		107. VMD. 2014. Responsible antibiotic use under the cascade. [Online]. Available from: https://assets.publishing.service.gov.uk/media/5a806fd8e5274a2e87db9ce6/646732_Responsible_Use_of_Antibiotics_on_the_cascade.pdf [Accessed 11th October 2023].
13	**Welsh Government (Animals and the Environment AMR Delivery Group)**	108. Welsh Government. 2019. Five Year Implementation Plan for Wales 2019-2024. [Online]. Available from: https://www.gov.wales/sites/default/files/publications/2019-07/antimicrobial-resistance-in-animals-and-the-environment-implementation-plan.pdf [Accessed 11th October 2023].
14	**World Health Organisation (WHO)**	109. WHO. 2017. Who Guidelines on use of Medically Important Antimicrobials in Food-Producing Animals. [Online]. Available from: https://iris.who.int/bitstream/handle/10665/258970/9789241550130-eng.pdf [Accessed 11th October 2023].
15		110. WHO. 2020. Antibiotic resistance. [Online]. Available from: https://www.who.int/news-room/fact-sheets/detail/antibiotic-resistance [Accessed 11th October 2023].
16	**World Organisation for Animal Health (OIE)**	111. OIE. 2015. Antimicrobial Resistance. [Online]. Available from: https://www.woah.org/app/uploads/2021/03/antibio-en.pdf [Accessed 11th October 2023].
17		112. OIE. 2015. Combating Antimicrobial Resistance and Promoting the Prudent Use of Antimicrobial Agents in Animals. [Online]. Available from: https://www.woah.org/fileadmin/Home/eng/Our_scientific_expertise/docs/pdf/AMR/A_RESO_AMR_2015.pdf [Accessed 11th October 2023].
18		113. OIE. 2016. The OIE Strategy on Antimicrobial Resistance and the Prudent Use of Antimicrobials. [Online]. Available from: https://www.woah.org/fileadmin/Home/eng/Media_Center/docs/pdf/PortailAMR/EN_OIE-AMRstrategy.pdf [Accessed 11th October 2023].
19		114. OIE. 2018. We need you to handle antimicrobials with care. [Online]. Available from: https://oie-antimicrobial.com/wp-content/uploads/sites/5/2018/12/EN-Leaflet-key-messages-181106-HDwcm.pdf [Accessed 11th October 2023].
20		115. OIE. 2018. Follow the Five ″Only″ Rules. [Online]. Available from: https://oie-antimicrobial.com/wp-content/uploads/sites/5/2018/10/Infographic-farmers-HD-WCM.pdf [Accessed 11th October 2023].
21		116. OIE. 2018. We need you to take action to ensure antimicrobials are handled with care. [Online]. Available from: https://oie-antimicrobial.com/wp-content/uploads/sites/5/2018/10/OIE-AMR-FARMERS-EN-HD-WCM.pdf [Accessed 11th October 2023].
22		117. OIE. 2020. OIE Standards, Guidelines and Resolutions on Antimicrobial Resistance and the use of antimicrobial agents. [Online]. Available from: https://www.woah.org/app/uploads/2021/03/book-amr-ang-fnl-lr.pdf [Accessed 11th October 2023].
23	**European Medicines Agency (EMA)**	118. EMA. Not Dated. Categorisation of antibiotics for use in animals for prudent and responsible use. [Online]. Available from: https://www.ema.europa.eu/en/documents/report/infographic-categorisation-antibiotics-use-animals-prudent-responsible-use_en.pdf [Accessed 11th October 2023].
24		119. EMA. 2016. Updated advice on the use of colistin products in animals within the European Union: development of resistance and possible impact on human and animal health. [Online]. Available from:

		https://www.ema.europa.eu/en/documents/scientific-guideline/updated-advice-use-colistin-products-animals-within-european-union-development-resistance-possible_en-0.pdf [Accessed 11th October 2023].
25		120. EMA. 2016. EMA and EFSA Joint Scientific Opinion on measures to reduce the need to use antimicrobial agents in animal husbandry in the European Union, and the resulting impacts on food safety (RONAFA). [Online]. Available from: https://www.ema.europa.eu/en/documents/report/ema-efsa-joint-scientific-opinion-measures-reduce-need-use-antimicrobial-agents-animal-husbandry_en.pdf [Accessed 11th October 2023].
26		121. EMA. 2019. Categorisation of antibiotics in the European Union. [Online]. Available from: https://www.ema.europa.eu/en/documents/report/categorisation-antibiotics-european-union-answer-request-european-commission-updating-scientific_en.pdf [Accessed 11th October 2023].
27		122. EMA. 2022. Reflection paper on prophylactic use of antimicrobials in 6 animals in the context of Article 107(3) of Regulation 7 (EU) 2019/6. [Online]. Available from: https://www.ema.europa.eu/en/documents/scientific-guideline/reflection-paper-prophylactic-use-antimicrobials-animals-context-article-1073-regulation-eu-2019/6_en.pdf [Accessed 11th October 2023].
**Food standards organisations & collaborations**		
1	**Codex Alimentarius (International Food Standards)**	123. Codex Alimentarius. 2021. Code of Practice to Minimize and Contain Foodborne Antimicrobial Resistance. [Online]. Available from: https://www.fao.org/fao-who-codexalimentarius/sh-proxy/en/?lnk=1&url=https%253A%252F%252Fworkspace.fao.org%252Fsites%252Fcodex%252FStandards%252FCXC%2B61-2005%252FCXC_061e.pdf [Accessed 11th October 2023].
2	**Food and Agriculture Organization of the United Nations (FAO)**	124. FAO. 2017. Handle Antimicrobials with Care. [Online]. Available from: https://www.fao.org/3/i8184e/i8184e.pdf [Accessed 11th October 2023].
3		125. FAO. 2021. The FAO Action Plan on Antimicrobial Resistance 2021–2025. [Online]. Available from: https://www.fao.org/3/cb5545en/cb5545en.pdf [Accessed 11th October 2023].
4	**Food Industry Initiative on Antimicrobials (FIIA)**	126. FIIA. 2021. FIIA Policy on Responsible Use of Antibiotics. [Online]. Available from: https://fiia.co.uk/wp-content/uploads/2021/08/FIIA-Policy-on-Responsible-Use-of-Antibiotics.pdf [Accessed 11th October 2023].
**Cross-sector working groups**		
1	**Alliance to Save Our Antibiotics**	127. Alliance to Save Our Antibiotics. 2017. Real farming solutions to antibiotic misuse. [Online]. Available from: https://www.saveourantibiotics.org/media/1777/asoa-report-real-farming-solutions-to-antibiotic-misues-what-farmers-and-supermarkets-must-do.pdf [Accessed 11th October 2023].
2		128. Alliance to Save Our Antibiotics. 2021. Antibiotic Use in Organic Farming Lowering Use Through Good Husbandry. [Online]. Available from: https://www.saveourantibiotics.org/media/1914/20210406_antibiotic_use_in_organic_farming.pdf [Accessed 11th October 2023].
